# Effectiveness of Participatory Ergonomic Interventions on Work-Related Musculoskeletal Disorders, Sick Absenteeism, and Work Performance Among Nurses: Systematic Review

**DOI:** 10.2196/68522

**Published:** 2025-06-18

**Authors:** Guganesan Krishnanmoorthy, Sanjay Rampal, Sumitra Ropini Karuthan, Faiz Baharudin, Rama Krishna

**Affiliations:** 1 Department of Social and Preventive Medicine, Faculty of Medicine, Universiti Malaya, Wilayah Persekutuan, Kuala Lumpur, 50603, Malaysia, 60 173625817; 2 Centre for Epidemiology and Evidence-based Practice, Department of Social and Preventive Medicine, Faculty of Medicine, Universiti Malaya, Kuala Lumpur, Malaysia; 3 Occupational Safety, Health and Environment Centre, Universiti Malaya, Kuala Lumpur, Malaysia; 4 Occupational Safety, Health and Environment Unit, Universiti Malaya Medical Centre, Kuala Lumpur, Malaysia; 5 Occupational Health Clinic, Universiti Malaya Medical Centre, Kuala Lumpur, Malaysia

**Keywords:** musculoskeletal disorders, nurses, sick absenteeism, work performance, ergonomics

## Abstract

**Background:**

Nurses face a higher risk of developing work-related musculoskeletal disorders (WMSDs) due to their primary roles in patient care. Participatory ergonomics (PE), an approach that integrates large-scale interventions performed at organizational and systems levels with small-scale interventions, is widely considered a promising approach to mitigate health problems at the workplace. However, its effectiveness in addressing WMSDs and secondary outcomes such as sickness absence and work performance among nurses is not fully understood.

**Objective:**

This systematic review assessed the effectiveness of PE interventions in preventing WMSDs and mitigating two related outcomes, sickness absence and work performance, among nurses.

**Methods:**

A literature search was performed in four electronic databases, PubMed, ScienceDirect, Scopus, and PsycNet, guided by the PRISMA (Preferred Reporting Items for Systematic Reviews and Meta-Analysis) guidelines to retrieve relevant papers published between 2017 and 2023. Papers fulfilling the eligibility criteria were analyzed and subjected to quality appraisal.

**Results:**

Overall, 19 papers were included in the final analysis. Various categories of ergonomic interventions were identified, with the predominant being exercise and physical activities, health promotional activities and training, educational programs, and patient handling devices. Multicomponent interventions, especially those involving physical activities and exercise, demonstrated stronger effects in reducing the risk of WMSDs at 6 months (OR 1.64, 95% CI 1.12‐4.54) and 12 months postintervention (OR 2.70, 95% CI 1.52‐4.51) compared with single interventions. However, most ergonomic interventions had no statistically significant effect (*P*>.05) on sickness absence and work performance. More than half (n=13) of the studies demonstrated moderate to high risk of bias, reflecting the need for better quality interventions.

**Conclusions:**

Multicomponent interventions, particularly those involving physical activities and exercise, are more effective in reducing the risk of WMSDs among nurses compared with individual interventions. However, their long-term effects in addressing WMSDs, sick absenteeism, and work performance are still unclear. These gaps could be addressed by integrating organizational factors and prevention policies into existing ergonomic interventions, thereby offering opportunities to improve psychological health, job satisfaction, and work dynamics.

## Introduction

### Background

Nurses are highly exposed to risks of work-related disorders and injuries, culminating in burnout and low quality of care [1]. Such injuries may involve all body organs, especially the musculoskeletal system [2]. Musculoskeletal disorders (MSDs) refer to health problems affecting the locomotor apparatus, such as the skeleton, muscles, tendons, cartilage, ligaments, and nerves [3]. MSDs comprise several degenerative and inflammatory conditions affecting the joints, tendons, ligaments, muscles, peripheral nerves, and supporting blood vessels, leading to soreness and physical discomfort [3].

Among health care workers, nurses have a higher risk of developing work-related musculoskeletal disorders (WMSDs) [4]. Nurses play pivotal roles within the health care system, which can be physically challenging as they frequently help patients to mobilize, transfer between positions, and execute other daily life activities [5]. Multiple factors have been found to increase the risk of WMSDs among nurses, including physical load and work posture [6], psychosocial factors such as the presence of psychosomatic symptoms and personality, work tasks, and work organizational factors [5]. Thus, it is not surprising that nursing staff stationed in the operating room recorded a higher prevalence of occupational injuries compared with intensive care nurses, nonspecialized nurses, and radiologic technicians [7]. Events including static body posture, prolonged standing, using tools such as retractors during surgery, and manual tasks such as pushing, pulling, and lifting patients and surgery sets could elicit discomfort and WMSDs, particularly among nurses working in operating units [8]. Recent studies have also highlighted factors such as low educational level, increasing age, high BMI, and lifestyle as predictors of WMSDs among nurses [4].

Given the aforementioned points, several authors have advocated and explored effective workplace interventions to address the risk of WMSDs among nurses. Occupation-specific interventions and rehabilitation options have the potential to reduce functional disabilities among nursing staff that may result from these injuries [8,9]. Ergonomic interventions, particularly participatory ergonomics (PE), are a promising approach to reducing WMSDs. PE integrates large-scale interventions that are performed at organizational and systems levels with small-scale interventions where workers are opportune to use their knowledge in addressing work-related ergonomic issues [8]. It entails the active involvement of workers in controlling and planning a significant amount of their tasks or work activities while introducing ergonomic knowledge, changes, and procedures in order to enhance working conditions, productivity, safety, quality, and comfort [9].

Positive outcomes such as lower musculoskeletal symptoms and occupational injuries, improved psychological health, as well as a decrease in workers’ compensation claims, productivity loss, and work absenteeism, were reported following PE interventions among various high-risk groups [9,10]. Despite ergonomic interventions being considered essential to reduce the increasing trend of WMSDs, there is no agreement or consensus on the most appropriate intervention, as the component and approach may vary with the workplace setting [[11]].

### Importance of Ergonomics in Nursing

The risk of WMSDs can be predicted based on ergonomic, individual, and psychosocial factors [8]. Ergonomics scientifically discusses how a job can be aligned with a person’s physical and psychological characteristics without causing any harm to the person’s well-being and efficiency [11]. The onset and development of WMSDs can be prevented by acquiring more knowledge and applying ergonomics [12]. Nurses are motivated to become more efficient, experience higher job satisfaction, and reduce job-related stress, occupational-related diseases, accidents, and absenteeism when they work in appropriate ergonomic conditions [13].

An earlier study revealed several advantages and enhanced occupational health among community nurses following the implementation of a multifaceted ergonomic program [14]. Moazzami et al [15] reported that alterations in nurses’ body movements were facilitated by ergonomic educational intervention, particularly from the contemplation and preparation phases to the action phase for adopting correct body postures in the operating room. Likewise, a significant reduction in musculoskeletal pain in the shoulder, neck, and knee regions was recorded among nurses in general wards after implementing an ergonomic educational intervention [16]. The use of ergonomic posture training among nursing assistants also led to a significant reduction in work-related low back pain 6 months after the intervention but did not affect disability scores [15]. However, some studies depicted limited success of ergonomic interventions on measured outcomes such as WMSDs [16,17], work productivity, and absenteeism [18], which may be linked to differences in the design of interventions, samples, data collection tools, settings, and research design. Thus, there is still limited information on the appropriate and effective ergonomic interventions for preventing and addressing WMSDs and their related consequences among nurses.

### Rationale for Conducting This Review

Although more research is required to develop conclusive evidence on the effectiveness of ergonomics interventions in reducing WMSDs among nurses, pertinent information is yet to be gleaned from the several available studies in the literature. In the last decade, numerous studies have demonstrated the importance of diverse programs such as participatory, educational, single, and multidisciplinary ergonomics interventions among nursing professionals [12,19,20]. Nevertheless, findings from these studies are yet to be systematically reviewed. Most of the systematic reviews focused on office workers or workplace settings [21] and dental and health care professionals [22]. However, Sun et al [23] in a recent systematic review and meta-analyses, compared the efficacy of nondrug interventions on nonspecific low back pain among nurses and found that the most effective intervention was a combination of low back exercises and health education, followed by single low back exercises and yoga.

Previous literature reviews have investigated the effectiveness of interventions among nurses and in nurses’ homes [14], including nursing assistants’ risk of handling obese and overweight patients and interventions to reduce low back pain [24]. However, none of the past reviews looked into how ergonomic interventions ameliorate WMSDs and their consequences, particularly among nurses. The present review differs from prior attempts by systematically analyzing the various ergonomic interventions adopted in preventing WMSDs and other closely related outcomes, including sickness absenteeism and work performance or productivity in nurses. It seeks to provide evidence-based data and scientific support for developing an ergonomic intervention to prevent WMSDs among nursing professionals.

The objective of this systematic review is to assess the effectiveness of ergonomic interventions in preventing WMSDs and mitigating two related outcomes, sickness absence and work performance, among nurses.

## Methods

### Overview

This systematic review was conducted according to the PRISMA (Preferred Reporting Items for Systematic Reviews and Meta-Analyses) statement. The PRISMA statement was developed to assist researchers in preparing a complete, accurate, and transparent review that is beneficial to users [25]. The PRISMA 2020 statement includes new reporting guidelines, particularly for systematic reviews of studies that appraise the effects of health interventions. The PRISMA statement was considered suitable for the present systematic review, given its provision for a clear definition of research questions, identification of the exclusion and inclusion criteria, and assessment of relevant and accessible scientific papers within a specific timeframe. In addition, this review has been registered in International Prospective Register of Systematic Reviews (PROSPERO) with the registration number CRD42023403981.

### Formulation of Research Questions

Formulation of a concise and clear research question is an important phase to guide the authors in many aspects of the systematic review. PICO, a research question development tool (RQDT), was used in the formulation of the research question for this review. PICO is an acronym in which P is population, I is intervention, C is comparison, and Ois outcome, and the 3 main concepts of PICO are population or problem, interest, and context. The authors of this review made an effort to incorporate all the aspects in synthesizing the main research question. Considering the population of interest as nurses, ergonomic intervention as the intervention, other nonergonomic interventions as the comparison group, and WMSDs, work performance, and sickness absenteeism as the outcomes, the research questions for this systematic review are as follows:

1. What are the commonly implemented participatory ergonomic interventions to prevent WMSDs among nurses?
2. Are ergonomic interventions effective in preventing WMSDs, sickness absenteeism and enhancing work performance among nurses?

### Systematic Literature Search

#### Identification

The identification process entailed the determination of suitable keywords to search for sufficient information. Aligning with the research question, a few main keywords were recognized, namely MSDs, WMSDs, musculoskeletal pain, and musculoskeletal symptoms. In terms of the study population, the selected keywords encompass nurse, nursing, nurse assistants, and health care professionalsor workers. While the former is more specific for the present study context, the term “health care workers” was considered since such studies may include diverse professionals including those from the nursing field. Moreover, by taking the term “health care” into account in the identified keywords, health care professions that entail heavy-lifting jobs (ie, important risk factors for WMSDs) can be covered in the literature search.

#### Search Strategy

Search functions, including truncation and Boolean operators (OR and AND), were used in combining the selected keywords, leading to the formation of search strings. Search strings relating to study participants or study population, intervention, and study outcomes were synthesized as follows:

1. Study participants: “Nurse” OR “Nursing” OR “Nurse assistant” OR “Healthcare professionals” OR “Healthcare workers.”
2. Intervention: “Ergonomic” OR “Ergonomic Intervention” OR “Intervention” OR “Participatory Ergonomics”
3. Outcomes: “Work-related musculoskeletal disorders” OR “Musculoskeletal disorder” OR “Musculoskeletal Pain” OR “Musculoskeletal symptom” OR “Sick Absence” OR “Sick leave” OR “Sick Absenteeism” OR “Work Performance” OR “Work Productivity.”

All the search strings outlined above were combined using the Boolean operator “AND,” leading to the final search string used for each database. However, since each database is unique and programmed to operate differently, the search strings were modified accordingly to suit each database and to maximize the number of hits. For instance, ScienceDirect only accepts a maximum of 8 search terms, while PubMed requires all keywords to be placed in parentheses.

A detailed literature search was then carried out independently by the researcher and a research collaborator in four electronic databases: PubMed, ScienceDirect, Scopus, and PsycNet. These databases were selected as they offer an extensive collection of relevant journals, provisions for customizing an advanced search, and storage of retrieved papers. The literature search was first conducted in March 2023 and updated in March 2024 to gather original research papers written in English and published between 2017 and 2023. The search string used in all 4 databases, the number of papers retrieved, and the date of acquisition are presented in Table 1.

**Table 1.** Search terms and the total number of papers retrieved from each database.

Search engines	Search string and search terms		Number of hits (N=5867)	Acquisition date
PubMed	Main search terms using document title, abstract, and keywords	(“Nurse” OR “Nursing” OR “Nurse assistant” OR “Healthcare professionals” OR “Healthcare workers”) AND (“Ergonomic” OR “Ergonomic Intervention” OR “Intervention” OR “Participatory Ergonomics”) AND (“work-related musculoskeletal disorders” OR “Musculoskeletal disorder” OR “Musculoskeletal Pain” OR “Musculoskeletal symptom” OR “Sick Absence” OR “Sick leave” OR “Sick Absenteeism” OR “Work Performance” OR “Work Productivity”)	525	March 4, 2024
Scopus	Main search terms using document title, abstract, and keywords	(“Nurse” OR “Nursing” OR “Nurse assistant” OR “Healthcare professionals” OR “Healthcare workers”) OR (“Ergonomic” OR “Ergonomic Intervention” OR “Intervention” OR “Participatory Ergonomics”) AND (“work-related musculoskeletal disorders” OR “Musculoskeletal disorder” OR “Musculoskeletal Pain” OR “Musculoskeletal symptom” OR “Sick Absence” OR “Sick leave” OR “Sick Absenteeism” OR “Work Performance” OR “Work Productivity”)	1009	March 5, 2024
ScienceDirect	Main search terms using document title, abstract, and keywords	(“Nurse” OR “Healthcare professional”) AND (“Ergonomic” AND “Intervention” OR “Participatory Ergonomics”) AND (“Musculoskeletal” OR “Sick” OR “Work Performance”)	1617	March 7, 2024
PsycNet	Main search terms using document title, abstract, and keywords	(“Nurse” OR “Nursing” OR “Nurse assistant” OR “Healthcare professionals” OR “Healthcare workers”) AND (“Ergonomic” OR “Ergonomic Intervention” OR “Intervention” OR “Participatory Ergonomics”) AND (“work-related musculoskeletal disorders” OR “Musculoskeletal disorder” OR “Musculoskeletal Pain” OR “Musculoskeletal symptom” OR “Sick Absence” OR “Sick leave” OR “Sick Absenteeism” OR “Work Performance” OR “Work Productivity”)	2716	March 8, 2024

#### Eligibility Criteria and Screening

Upon completing the literature search, the retrieved papers were screened according to the eligibility criteria. The first screening process was performed by using the filter feature provided in all 4 search engines. In terms of type of publication, only original papers were selected for further review, whereas chapters in books, books, and conference proceedings were removed. As for review papers, only those relevant to our review objective were assessed for vital information and to identify research papers that might have been missed during the literature search.

In terms of study duration, only papers in the last 7 years were considered for full-text reading. Based on preliminary analysis, the proposed timeline is sufficient to yield papers to facilitate a representative and comprehensive review of the research topic. Finally, only papers written and published in English were included in this study. This decision was taken to prevent any challenges during the data extraction since the researcher is only proficient in English. Issues arising during the process were resolved by consensus between all the review authors.

#### Data Extraction and Analysis

All the final papers were subjected to qualitative analysis. An Excel (Microsoft) spreadsheet form was generated before reviewing the papers to document basic data from the studies, such as the authors’ names, titles, journals, and publication years. Data were extracted from each paper by scanning through the abstracts, methods, and results before proceeding to the full text. These processes assisted the researcher in recording the year of publication, study location, study design, specific ergonomic intervention used, instrument used, and main results or emerging themes.

First, available data were analyzed thematically to generate themes and subthemes. The potential themes considered were as follows: (1) study characteristics, (2) quality assessment, (3) common types of participatory ergonomic interventions, (4) multicomponents versus single components, and (5) effects of participatory ergonomic interventions on WMSDs, sickness absence, and work performance. Contrast analyses, specifically pairwise comparisons, were performed to ascertain the effect size report in each study involving intervention and control groups. For studies reporting WMSDs as categorical outcomes, the odds ratio (OR, 95% CI) was computed to measure the strength and direction of the association. Meanwhile, the mean difference (95% CI) was computed for studies reporting the outcome variables as continuous outcomes.

#### Quality Appraisal

Quality appraisal of each paper was performed using the widely accepted quality assessment tools for intervention studies, the *Cochrane Handbook for Systematic Reviews of Interventions* and the Risk of Bias in Non-randomized Studies-of Interventions (ROBINS-I) tool [26,27]. The Cochrane tool was used for experimental studies reporting randomized interventions.

A total of 5 domains were considered for explicit reporting, comprising the sequence generation, allocation concealment, blinding, and completeness of data. For the first domain, the use of a randomized sampling method was assessed either by a random number table, tossing a coin, or computerized-generated numbers. Regarding the blinding of recruited patients, studies were considered adequate if both the intervention providers and outcome assessors were blinded (ie, minimal risk of compromised blinding) and inadequate if no blinding or partial blinding was performed. For outcome measures, trials were recorded as adequate if there were no missing data or if they stated the reasons for missing data that were unlikely to be associated with the true outcome. In addition, the studies were evaluated for selective reporting. Studies without sufficient information regarding the outcome reporting process were considered unclear. For all the aforementioned criteria, studies with insufficient information about each criterion were considered unclear. Other potential sources of bias were assessed, such as demographic characteristics between the treatment groups, by considering the baseline information of the participants.

Using the ROBINS-I tool, similar procedures described above were applied for the nonrandomized studies. The quality assessment tool focused on the randomization method, blinding of drug administration and assessment of treatment outcomes, data analysis techniques, sample size, and steps taken in addressing missing data and loss to follow-up. Studies reporting at least 70% (8/12) of the items in the quality assessment tool were considered of higher methodological quality or low risk of bias [27].

### Ethical Considerations

Systematic review is part of a module development.Module development has obtained study ethics approval from the Medical Research Ethical Committee of Universiti Malaya Medical Centre (MREC ID No:20221019-11632) and National Medical Research Register (NMRR ID-23-01804-F44).

## Results

### Search Results

**Figure 1.**
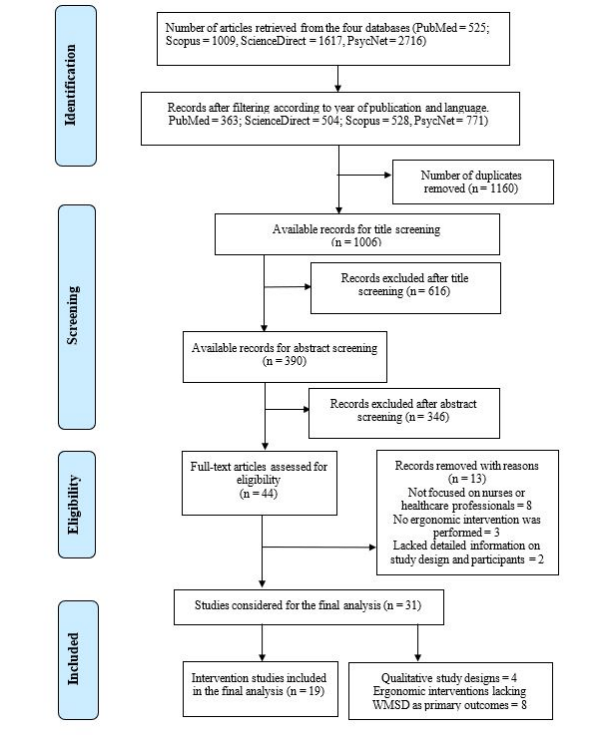
Literature search process using the Preferred Reporting Items for Systematic Reviews and Meta-Analyses guidelines. WMSD: work-related musculoskeletal disorder.

The initial search process before filtering and screening resulted in a total of 5867 papers, comprising 525 from PubMed, 1009 from Scopus, 1617 from ScienceDirect, and 2716 from PsycNet (Figure 1). Upon considering the years of publication (2017‐2023) and language (ie, English), the remaining papers were reduced to 2166 (ie, PubMed=363, ScienceDirect=504, Scopus=528, and PsycNet=771).

Thereafter, 1160 papers were identified as duplicates and removed accordingly. To further reduce the number of records, the remaining results from each database were sorted and subjected to title screening. Out of the remaining papers (n=1006), those with irrelevant titles (n=616) and abstracts (n=346) were removed, leading to a total of 44 papers for full-text reading. Around 25 studies were excluded after reading the full texts because of irrelevant study populations (ie, not involving nurses or health care professionals, n=8), not reporting any ergonomic intervention (n=3), unclear study design (n=2), qualitative study designs (n=4), and ergonomic interventions with WMSDs as primary outcomes (n=8).

### Descriptive Findings

Overall, 19 papers were experimental studies involving ergonomic interventions among nurses and other related health care professionals. These papers reported at least one of the following outcomes: WMSDs symptoms or pain, sickness absence, and work performance. Hence, all 19 papers were included in the final analysis and systematically reviewed. Table S1 in Multimedia Appendix 1 depicts the studies and participants’ characteristics of types of ergonomic interventions and a summary of the main findings.

### Ergonomic Interventions Reported in the Papers

As shown in Table 2, 7 categories of ergonomic interventions were identified in the 19 studies as follows: (1) exercise and physical activities, (2) educational programs, (3) health promotional activities and training, (4) patient handling devices or equipment (acquired ergonomics equipment), (5 programmes) workstation and administrative interventions (organizational management), (6) counseling, and (7) others (case management and transcutaneous electrical nerve stimulation). The predominant ergonomic intervention was exercise and physical activities (11/19, 58%), followed by health promotional activities and training (8/19, 42%), educational programs (7/19, 36%), patient handling devices (4/19, 21%), workstation and administrative interventions (n=3), counseling (n=3), and case management (n=2). Furthermore, 3 studies reported specific exercises for back pain, 2 for neuromuscular pain, and 1 for neck pain. Around 10 studies (53%) each entailed either only multicomponent or single intervention, whereas 1 study compared both types of interventions.

**Table 2.** Types of participatory ergonomic interventions applied in the 19 studies.

Reference	Educational ergonomics	Counseling	Patient handling device or ergonomics equipment	Health promotion activities	Workstation adjustment and administrative	Exercise and physical activities	Others (social media/website, case management, nerve stimulation, and online apps)
Abdollahi et al [8]	P^a^	A^b^	A	A	A	A	A
Jalalvandi et al [16]	A	A	P	A	A	P	A
Soler-Font et al [19]	A	A	A	P	A	P	P
Yang et al [28]	P	A	A	P	A	A	A
Rasmussen et al [10]	A	A	A	P	A	P	A
Nguyen et al [29]	A	A	A	P	A	P	A
Pereira et al [30]	A	A	A	P	P	P	A
Chaiprateep et al [31]	A	A	A	A	A	P	A
Suni et al [32]	A	P	A	A	A	P	A
Higuchi et al [33]	P	P	A	A	A	A	A
Jakobsen et al [34]	P	A	A	A	A	A	A
Al-Quasi et al [35]	A	A	P	A	A	A	A
Sezgin and Esin [36]	P	A	A	A	A	A	A
Beyan et al [17]	A	P	P	P	P	A	A
Wulff Risør et al [37]	A	A	P	A	A	A	A
Imai et al [38]	P	A	A	A	A	P	A
Marshall et al [39]	P	A	P	A	P	A	A
Taulaniemi et al [40]	A	A	A	A	A	P	A
Hosseini et al [41]	A	A	A	P	A	A	A

a P: present.

b A: absent.

c NA: not available.

### Work-Related Musculoskeletal Disorders and Pain

#### Single Interventions

Among the 10 papers involving a single ergonomic intervention, significant reductions in either the prevalence, frequency, or pain intensity of WMSDs were reported in 6 studies [8,31,34,36,40,41]. The educational programs delivered by experts on ergonomic principles and risk factors of WMSDs led to a significant decrease (*P*<.05) in the risk of WMSDs, particularly in the ankle, wrist, lower back, and neck, pain intensity and Rapid Upper Limb Assessment ergonomic risk scores among nursing personnel with similar low effect sizes in both studies (0.30 vs 0.40) [8,36]. Likewise, ergonomic exercise (ie, neuromuscular and back exercise) performed in 3 studies was associated with significantly lower scores for low back pain (*P*=.05, effect size=0.45) [40] and overall pain scores (effect size=0.30‐0.52) compared with either a control group or other nonergonomic single intervention such as transcutaneous electrical nerve stimulation [16,31].

In addition, 2 studies involving single ergonomic interventions, particularly online applications, were identified in this review [8,33]. Both studies recorded significantly lower low back pain scores in the intervention group compared to the control, with moderate to low effect size (ie, 0.30‐0.50). Higuchi et al [33] focused on individual online therapy for low back pain, whereas Hosseini et al [41] used a Nursing Stretch Break app and reflected a significant reduction in WMSDs symptoms and pain intensity in nurses’ body parts except the elbows and knees.

In contrast, 3 of the remaining studies found no significant effects of single ergonomic interventions such as educational programs [34], individual-based interventions [17], and patient handling devices [37] in preventing or managing WMSDs among nursing personnel. In Jakobsen et al [34] and Risør et al [37], despite improvement in the usage of patient-assisted devices posteducational program, no significant change was observed in the risk of low back pain and other WMSDs at 12-month follow-up. Similarly, individual-based ergonomic programs, including training, engineering, and administrative interventions, did not affect WMSDs scores at 18 months follow-up [17].

#### Multicomponent Interventions

Among the studies involving multicomponent ergonomic interventions, despite several combinations of interventions being reported, the results were analyzed based on whether they included exercise or physical activities or health promotional activities.

Around, 3 studies entailed a combination of health promotion activities and case management [19], workstation adjustment, and training without performing any exercise or physical activities [18,28]. Results from the studies revealed a significant reduction in the risk of WMSDs with an OR ranging from 1.64 (95% CI 1.12‐4.54) at 6 months postintervention to 2.70 (95% CI 1.52‐4.51) at 12 months postintervention [19,28]. In contrast, Rasmussen et al [10] found no significant effect of an intervention comprising three training workshops centred on focusing on workstation adjustment and health promotion in addressing WMSDs.

As for the 3 multicomponent interventional studies involving physical exercise, the pain intensity arising from WMSDs such as the lower back and shoulder or upper arm, neck, and hand or wrists body regions reduced significantly either 6 months or 12 months postintervention [28,32,38]. The effect size reported in these studies ranged from moderate to high (0.54‐0.80), reflecting the greater efficacy of multicomponent PE comprising general or specific body exercise, for the management of WMSDs in nurses. Notably, the strongest effect size was observed in studies whereby exercise or physical activities were combined with either health promotion activities or educational or counseling programs such as pain neuroscience education and back care counseling [32,38]. Finally, the study by Marshall et al [39] found a significantly moderate reduction (OR=0.40) in subsequent acute cases of WMSD following multifactorial interventions comprising equipment intervention, educational ergonomics, and no-lift policies (administrative interventions).

#### Work Performance and Related Outcomes

Only 4 of the 19 papers considered work performance or presenteeism as musculoskeletal-related work performance, with 3 comprising multicomponent [19,30,38] and 1 including only a single intervention [40]. Both Imai et al [38] and Pereira et al [30] performed a dual-component intervention (ie, exercise and Pain Neuroscience Education (PNE) and workstation ergonomics and neck-specific exercise), leading to significant improvements in presenteeism (moderate to strong effect size=0.52‐0.78) at 12 months follow-up. Meanwhile, using the same follow-up period, Soler-Font et al [19] found no significant difference in work functioning and performance in nurses exposed to a combination of PE, health promotion activities, and case management relative to the control group.

For the single-component studies, Taulaniemi et al [40] revealed a significant reduction in work-interfering pain (effect size=0.40; *P*=.046) in heavy nursing duties. Suni et al [32] also depicted that operating room nurses subjected to only back care counseling or neuromuscular exercise had a higher risk (OR 0.46) of work-related fear of pain compared with those administered a combination of both ergonomic interventions.

#### Sick Absenteeism

Sick absenteeism was reported in 6 studies included in this review, with 3 reporting multicomponent [10,19,30], 2 involving only single ergonomic interventions [17,32,37], and 1 study comparing both types [32].

For the multicomponent interventions, 2 studies found a significant reduction in musculoskeletal-related sickness absence in the intervention relative to comparison groups at 20 weeks [10] and 12 months postintervention [30]. Specifically, Rasmussen et al [10] entailed 20 weeks of PE comprising training workshops and health promotion activities, whereas Pereira et al [30] compared nurses subjected to workstation ergonomics and neck-specific exercises to those exposed to health promotion activities or information. Both studies found a moderate to strong effect size (−0.48 days per month, 95% CI −0.8 to −0.10 vs −0.72 days per month, 95% CI −0.52 to −0.94). In contrast, no significant difference was detected in sickness absence between nurses who administered PE, health promotion activities, and case management compared with the control at 12 months postintervention [19].

The 2 studies involving single interventions, such as individual-based training, stretching exercises, motivation meetings, and patient handling devices or equipment, found no significant effect on sickness absence among nurses at 12 months [37] and 18 months of foled a singlelow-up [17]. These studies acknowledged the low likelihood of single-component ergonomic interventions in addressing musculoskeletal-related sickness absence, thus advocating for multicomponent programs, particularly administrative measures. This was further evidenced in the study comparing 4 different intervention arms, whereby only the combined arm (ie, neuromuscular exercise and back care counseling) demonstrated a significantly lower mean number of sickness absence days (effect size=0.55, *P*=.025) relative to the exercise-only groups at 12-month follow-up.

#### Quality Assessment Results

Out of the 11 RCTs evaluated using the Cochrane tool, 6 studies demonstrated a low risk of bias [10,19,28,30,32,34], 3 recorded a moderate risk of bias [16,33,40], and 2 papers had a high risk of bias [39,42], as shown in Table 3. All 6 studies with a low risk of bias showed high methodological quality in terms of randomization methods, concealment of treatment allocation, no significant difference between treatment groups at baseline, intervention delivery and adherence, as well as accounting for missing data and adjustment for possible confounders. Only one of the 6 studies performed a single-blinding procedure for the outcome assessment [32]. Meanwhile, the main issues observed in studies with moderate to high risk of bias were unclear or lack of information on the participants’ baseline characteristics (n=4), a strong potential for confounding of the intervention’s effect (n=4), lack of appropriate analysis method for confounder adjustment (n=5), no information on how missing data were addressed (n=3), incoherent onset of intervention and follow-up in most participants (n=3), unclear definition of intervention groups (n=2), poor adherence to the intervention regimen (n=2), and indications of selective reporting (n=2).

**Table 3.** Risk of bias assessment of the experimental studies using the Cochrane tool (n=11).

Items	Jalalvandi et al [16]	Soler-Font et al [19]	Yang et al [28]	Rasmussen et al [10]	Pereira et al [30]	Suni et al [32]	Higuchi et al [33]	Jakobsen et al [34]	Imai et al [38]	Marshall et al [39]	Taulaniemi et al [40]
Bias due to confounding											
Is there potential for confounding of the effect of the intervention in this study?	N^a^	N	N	N	N	N	N	N	Y^b^	Y	N
Was the analysis based on splitting participants’ follow-up time according to the intervention received?	Y	Y	Y	Y	Y	Y	Y	Y	Y	N	Y
Were intervention discontinuations or switches likely to be related to factors that are prognostic for the outcome?	NA^c^	N	N	N	NA	NA	NA	NA	N	Y	NA
Baseline confounding and time-varying confounding											
Did the authors use an appropriate analysis method that controlled for all the important confounding domains?	Y	Y	Y	NA	NA	Y	Y	Y	Y	N	Y
Did the authors control for any postintervention variables that could have been affected by the intervention?	N	Y	N	Y	Y	Y	N	Y	Y	N	N
Did the authors use an appropriate analysis method that controlled for all the important confounding domains and for time-varying confounding?	Y	Y	Y	NA	NA	Y	Y	Y	Y	Y	Y
Bias in selection of participants for the study											
Was selection of participants into the study (or into the analysis) based on participant characteristics observed after the start of the intervention?	N	N	N	NA	N	N	N	Y	N	N	N
Do the start of follow-up and the start of intervention coincide for most participants?	Y	Y	Y	Y	Y	Y	Y	Y	N	N	Y
Bias in classification of interventions											
Were intervention groups clearly defined?	Y	Y	Y	Y	Y	Y	Y	Y	Y	N	Y
Was the information used to define intervention groups recorded at the start of the intervention?	Y	Y	N	Y	N	Y	Y	Y	Y	N	Y
Could classification of intervention status have been affected by knowledge of the outcome or risk of the outcome?	N	N	N	N	N	N	N	N	N	Y	N
Bias due to deviations from intended interventions											
Were there deviations from the intended intervention beyond what would be expected in usual practice?	NA	N	N	N	N	N	N	N	N	NA	NA
Effect of starting and adhering to intervention											
Were important cointerventions balanced across intervention groups?	N	Y	Y	Y	Y	Y	Y	Y	Y	N	N
Was the intervention implemented successfully for most participants?	NA	Y	Y	Y	Y	Y	Y	Y	Y	Y	NA
Did study participants adhere to the assigned intervention regimen?	NA	NA	NA	Y	NA	NA	Y	NA	Y	Y	NA
Bias due to missing data											
Were outcome data available for all, or nearly all, participants?	Y	N	Y	Y	Y	Y	Y	Y	Y	Y	Y
Were participants excluded due to missing data on intervention status?	Y	N	N	N	N	N	Y	NA	NA	Y	Y
Were participants excluded due to missing data on other variables needed for the analysis?	N	N	N	N	N	N	Y	NA	N	Y	N
Bias in measurement of outcomes (blinding)											
Could the outcome measure have been influenced by knowledge of the intervention received?	N	N	N	N	N	N	N	N	Y	N	N
Were outcome assessors aware of the intervention received by study participants?	N	N	Y	N	Y	Y	Y	Y	N	Y	N
Were the methods of outcome assessment comparable across intervention groups?	Y	Y	Y	Y	Y	Y	Y	Y	Y	N	Y
Were any systematic errors in measurement of the outcome related to intervention received?	N	N	N	N	N	N	N	N	N	N	N
Is the reported effect estimate likely to be selected based on the result?											
Multiple outcome measurements within the outcome domain?	N	N	N	Y	N	N	N	N	N	N	N
Multiple analyses of the intervention-outcome relationship?	N	N	N	N	N	N	N	N	N	N	N
Different subgroups?	N	N	N	Y	N	N	N	N	N	N	N
Overall risk of bias grade	Moderate	Low	Low	Low	Low	Low	Moderate	Low	High	High	Moderate

a N: no.

b Y: yes.

c NA: not available.

Table 4 depicts the quality appraisal for the nonrandomized or quasi-experimental studies (n=8), with 6 [8,17,31,35-37], and 2 papers identified as having high risk and moderate risk of bias, respectively [17,29]. The main methodological weaknesses observed in these studies were unclear or lack of information on the participants’ baseline characteristics (n=3), the potential for confounding of the intervention’s effect (n=3), limited information on missing data analysis (n=5), unclear definition of intervention groups (n=4), poor adherence to the intervention regimen (n=3), low sample size for acceptable statistical power (n=3), lack of appropriate analysis method for confounder adjustment (n=6), and disparity in the onset of intervention, and follow-up in most participants (n=4). In addition, 4 of the studies did not report the measures taken to ensure consistency in intervention delivery, and only 2 reported measures of intervention adherence. None of these studies performed a blinding procedure, as the active treatment group was usually recognisable.

**Table 4.** Risk of bias assessment of the experimental studies using the Risk of Bias in Non-randomized Studies - of Interventions tool (n=8).

Items	Chaiprateep et al [31]	Abdollahi et al [8]	Nguyen et al [29]	Al-Qaisi et al [35]	Sezgin and Esin [36]	Beyan et al [17]	Risør et al [37]	Hosseini et al [41]
Bias due to confounding								
Is there potential for confounding of the effect of intervention in this study?	Y^a^	Y	N^b^	Y	Y	N	Y	N
Was the analysis based on splitting participants’ follow up time according to intervention received?	Y	N	Y	Y	N	Y	N	Y
Were intervention discontinuations or switches likely to be related to factors that are prognostic for the outcome?	N	Y	NA^c^	Y	Y	NA	Y	NA
Baseline confounding and time-varying confounding								
Did the authors use an appropriate analysis method that controlled for all the important confounding domains?	Y	N	Y	Y	N	Y	N	Y
Did the authors control for any post-intervention variables that could have been affected by the intervention?	Y	N	Y	N	N	Y	N	N
Did the authors use an appropriate analysis method that controlled for all the important confounding domains and for time-varying confounding?	Y	Y	Y	N	Y	Y	Y	Y
Bias in selection of participants into the study								
Was selection of participants into the study (or into the analysis) based on participant characteristics observed after the start of intervention?	N	N	Y	N	N	N	N	N
Do start of follow-up and start of intervention coincide for most participants?	N	N	Y	N	N	Y	N	Y
Bias in classification of interventions								
Were intervention groups clearly defined?	Y	N	Y	Y	N	Y	N	Y
Was the information used to define intervention groups recorded at the start of the intervention?	N	N	Y	Y	N	Y	N	Y
Could classification of intervention status have been affected by knowledge of the outcome or risk of the outcome?	Y	Y	N	N	Y	N	Y	N
Bias due to deviations from intended interventions								
Were there deviations from the intended intervention beyond what would be expected in usual practice?	NA	NA	NA	N	NA	NA	NA	NA
**Effect of starting and adhering to intervention**								
Were important co-interventions balanced across intervention groups?	NA	NA	N	Y	N	N	N	N
Was the intervention implemented successfully for most participants?	Y	NA	NA	Y	Y	NA	Y	Y
Did study participants adhere to the assigned intervention regimen?	NA	NA	Y	Y	Y	Y	Y	NA
Bias due to missing data								
Were outcome data available for all, or nearly all, participants?	Y	Y	Y	Y	Y	Y	Y	Y
Were participants excluded due to missing data on intervention status?	NA	N	Y	NA	Y	Y	Y	Y
Were participants excluded due to missing data on other variables needed for the analysis?	N	N	Y	N	Y	Y	NA	NA
Bias in measurement of outcomes (blinding)								
Could the outcome measure have been influenced by knowledge of the intervention received?	Y	Y	Y	Y	Y	Y	Y	N
Were outcome assessors aware of the intervention received by study participants?	N	Y	N	N	Y	N	Y	N
Were the methods of outcome assessment comparable across intervention groups?	N	N	Y	Y	N	Y	N	Y
Were any systematic errors in measurement of the outcome related to intervention received?	N	N	N	N	N	N	N	N
Is the reported effect estimate likely to be selected on the basis of the result?								
Multiple outcome measurements within the outcome domain?	N	N	N	N	N	N	N	N
Multiple analyses of the intervention-outcome relationship?	N	N	N	N	N	N	N	N
Different subgroups?	N	N	N	N	N	N	N	N
Overall risk of bias	High	High	Moderate	High	High	Moderate	High	High

a Y: yes.

b N: no.

c NA: not available.

## Discussion

### Principal Findings

Despite the heterogeneity of the 19 studies in this review in terms of study design, interventions used, sample size, measured outcomes, and research instruments, we were able to identify the participatory ergonomic interventions designed specifically for nursing professionals. In addition, the research designs and results revealed the methodological quality of the studies and evidence of the interventions in ameliorating WMSDs among this high-risk group of health care workers.

Descriptive findings from the systematic review revealed that the predominant PE interventions, specifically for preventing and managing WMSDs among nurses, were physical activities or exercise, health promotional activities, educational programs, and patient handling devices. These findings are consistent with previous systematic and integrative reviews focusing on interventions to address WMSDs and their secondary outcomes among health care professionals [43,44]. Physical activities and specific exercises are frequently considered in PE interventions either as individual or multifaceted approaches, given their efficacy in reducing musculoskeletal pain and associated symptoms [44]. Educational programs and health promotional activities are also integrated with most multicomponent interventions to enhance nurses’ knowledge, attitude, and self-efficacy toward lifestyle changes and behaviors to mitigate risk factors of WMSDs [43,45].

Single or individual interventions were applied in 10 studies ranging from physical activity to educational programs and the use of patient handling devices. Nevertheless, contradictory findings were gleaned from the studies, with few reflecting a low to moderate reduction in WMSD risk and ergonomic risk scores following educational programs [8,36]. Ergonomic exercise (ie, neuromuscular and back exercise) conducted in 3 studies also led to significantly lower scores for low back pain [40] and overall body pain scores [16,31]. These findings corroborate reports of individual exercise and physical activity proposed by Jakobsen et al [34] for the generality of health care professionals. Several prior studies have equally emphasized the positive effect of physical exercise on MSDs [20,29]. Moreover, physical exercise helps in preventing recurring and new episodes of WMSD pain [46], improving endurance, strength, and neuromuscular control in rehabilitation patients [47]. In contrast, 3 studies found no significant preventive effects of single interventions such as educational programs, individual-based interventions, and patient handling devices in ameliorating WMSDs among nurses and health care professionals [17,34,37]. These studies reflect the need for a multifaceted approach to addressing musculoskeletal pain, especially when designing interventions that include educational ergonomics. In other words, rather than using educational programs in isolation, they should be combined with other interventional approaches such as exercise.

As observed in this review, several studies entailed multifactorial interventions—a combination of procedures considering numerous factors and events acting on multiple levels [48]. This approach aligns with the multifaceted causes of WMSDs [19], which require integrated and comprehensive management strategieseducational program rather than isolated approches [42]. Comparisons between multicomponent and single interventions depicted that the former was more effective in reducing the risk of WMSDs among nurses relative to single type of ergonomic interventions. These results were evidenced in several studies involving a combination of health promotion activities and case management [19], workstation adjustment and training [10,28], educational ergonomics and patient handling equipment [39], as well as those involving physical activities and exercise [29,32,38]. In addition, moderate to high effect sizes were reported in studies where multicomponent interventions included at least one or more physical activities and exercise, whereas effects in those without exercise were generally low to moderate. These findings are consistent with a recent review conducted among health care professionals, in which the strongest reduction in WMSD injuries and pain stemmed from combined manual or mechanical patient lifting and handling training [44]. This could be explained by the fact that exercise, patient handling devices, and ergonomic training offer opportunities to mitigate physical risk factors and insufficient handling knowledge, which are the main contributing events to injuries among nurses and health care professionals [49,50]. More importantly, these studies highlight the significance of multifaceted PE aimed at comprehending the appropriate handling of patients or using the principles of biomechanics in executing such activities [30] and guidelines implementation [18]. These findings align with the Occupational Safety and Health Administration recommendations for increased availability of assistive facilities and devices, including both sophisticated and basic devices, that are crucial for safe patient handling [40].

Only a few reviewed studies investigated work performance and sickness absence, which may be because these events are secondary outcomes of WMSDs. This finding is not surprising, given that a systematic review of RCTs aimed at reducing sickness absence among health care workers identified only 7 relevant papers [45]. Results from the present review highlight the need to further explore how nurses’ work performance and sickness absence can be improved by ergonomic interventions. Most studies found no significant effects of multicomponent and individual interventions on these secondary outcomes at different follow-up points [19,28,32]. For instance, complaints of low back pain and its associated cost persisted among nurses for several weeks and months after performing neuromuscular exercise and back care counseling [32].

These studies raise the question of whether some multifaceted PE interventions, such as ergonomic education, health promotion activities and case management, neuromuscular exercise, and back care counseling, are either productive or counterproductive in improving secondary outcomes primarily linked to WMSDs among nurses. On the contrary, reduced work-day absences and increased safe patient handling were observed in health care professionals subjected to task-specific and multifactorial interventions such as mechanical and practical in-ward activities [50]. These promising results from studies conducted among health care workers emphasized changing the work dynamics, increasing job satisfaction, productivity, and the complicity between workers [32]. Moreover, psychological stress constitutes approximately 28% and 84% of upper back injuries in health care professionals [51].

Although qualitative studies were not included in this review, results from the studies highlighted the importance of considering psychological health problems, ergonomic knowledge, and preventive behaviors that could shape the risk of WMSDs, sickness absence, and work performance [11,18]. Organizational, physical, psychosocial, and personal factors were found to play pivotal roles in the development of musculoskeletal problems and their related consequences, thereby supporting the use of multifaceted interventions. Hence, as recommended by Carneiro et al [52], the organizational aspects of the working environment have to be considered in order to address WMSDs and their direct consequences on nurses’ health and productivity.

Multiple approaches are required to effect changes in practices and encourage a safety culture, such as ongoing training, skills, workflow processes, supervision, and communication between health care professionals about the risks [43,44]. This diverse and multifaceted approach is also crucial considering that long-term adherence to interventions is necessary for sustainable positive impacts on WMSDs, especially their secondary outcomes. For instance, although nurses enduring from WMSDs can experience significant relief following a short-term ergonomic intervention, a prolonged duration is required for the effects on sick leaves and performance [45]. Hence, the long-term beneficial effects of adhering to these multicomponent interventions need to be analyzed and relentless efforts to effect the necessary changes are equally important [41].

Quality assessment results underscored various areas to be considered when interpreting the systematic review findings. For instance, moderate and high risk of bias was observed in 5 and 8 studies, respectively. Thus, only 6 papers were of high quality. Methodological issues identified in the studies mainly entailed limited information on participants’ baseline characteristics, potential confounders that may affect the intervention’s effect, not addressing missing data, incoherent onset of intervention and follow-up, and poor adherence to the intervention regimen. These findings highlight the need for well-designed and high-quality RCTs.

The strength of the systematic review stems from being among the first attempts to gather current evidence on ergonomic interventions for nursing professionals and their effects on WMSDs, sickness absence, and work performance. In addition, a comprehensive and systematic approach was used, thereby assisting the researcher in retrieving relevant papers from selected databases. Quality appraisal was also performed to identify the methodological gaps that need to be bridged in future research. Nevertheless, the limitations are well acknowledged. Given that only papers published between 2015 and 2023 were considered in the review, papers published before these dates might contain important findings that are relevant to the research topic. The literature search and screening processes were performed only by the principal investigator, reflecting the possibility of bias and unknown intraobserver reliability. Moreover, only papers written in English in 4 databases were considered, thus limiting relevant studies written in other languages that might have assisted in answering the review questions.

### Conclusions

This systematic review synthesized evidence published between 2017 and 2024 on ergonomic interventions to reduce WMSDs and sickness absence and improve work performance among nurses. The most common interventions entailed physical activities or exercise, educational programs, health promotional activities, and training on the usage of patient handling devices or equipment, which were usually applied either as a single or multicomponent approach. Overall, the findings suggest that multifaceted interventions are more effective in reducing the risk of WMSDs and the associated pain in nursing professionals compared with individual interventions. The strongest effect size was observed in multicomponent interventions comprising at least one or more physical activities and exercise. Although these positive results are expected to lead to improved work functioning, work performance, and reduced sick leaves, available results are convincing to support the use of either single or combined ergonomic programs in achieving these secondary outcomes.

Apart from conducting high-quality RCTs, addressing the identified research gaps requires the integration of organizational factors and prevention policies to enhance psychological health, job satisfaction, and work dynamics into multicomponent ergonomic interventions. Since most interventions are designed for short-term periods, efforts to ensure their long-term effects are crucial for sustainable positive impacts on WMSDs, sickness absence, and work performance. For instance, introducing curricula content on the management of WMSDs and improving work performance, sickness absence and psychological health in nursing education may assist nurses in adopting the measures discussed in this review in clinical practice. This review may guide health care institutions on the most appropriate ergonomic interventions to prevent or reduce WMSDs among nurses, given their significance for well-being and economic implications in terms of absenteeism, work performance, and care for clients enduring mobility problems.

## Supplementary material

10.2196/68522 Multimedia Appendix 1 Descriptive characteristics of the studies included in this systematic review and analysis (n = 19).

10.2196/68522 Checklist PRISMA (Preferred Reporting Items for Systematic Reviews and Meta-Analyses) checklist.

## Abbreviations

MSD: musculoskeletal disorder
OR: odds ratio
PE: participatory ergonomics
PICO: Population, Intervention, Comparison, and Outcome
PRISMA: Preferred Reporting Items for Systematic Reviews and Meta-Analyses
PROSPERO: International Prospective Register of Systematic Reviews
WMSD: work-related musculoskeletal disorder
