# Supplementary material for: Effectiveness of Participatory Ergonomic Interventions on Work-Related Musculoskeletal Disorders, Sick Absenteeism, and Work Performance Among Nurses: Systematic Review
Source: JMIR Hum Factors. 2025 Jun 18;12:e68522. doi: 10.2196/68522 (PMC12192185; doi:10.2196/68522)
Supplement: Multimedia Appendix 1 [file humanfactors-v12-e68522-s001.docx]

**Supplementary Table 1:** Descriptive characteristics of the studies included in this systematic review and analysis (n = 19)

| S/N | References | Aim/objectives | Study design | Targets/participants | Outcomes | Type of intervention | Intervention | Findings |
| --- | --- | --- | --- | --- | --- | --- | --- | --- |
| 1 | Abdollahi et al. (2020) (8) | Ergonomics educational programme | Quasi-experimental | Nursing staff working in the operating room (37 nurses each in the intervention and control groups from 2 hospitals) in Iran | Prevalence and risk of Musculoskeletal disorders | Single | Ergonomics educational programme was performed and assessed biweekly for three months. The educational sessions covered several topics on MSDs epidemiology. | The intervention group recorded an overall decrease in the risk of MSDs post-educational programme (*p* = 0.03), particularly in the ankle, wrist, low back, and neck. |
| 2 | Rasmussen et al. (2018) (10) | Training for a participatory ergonomic intervention | 2-arm CRCT | Childcare nursing staff (n = 190 participants from 16 childcare institutions)  Denmark | Primary outcomes: pain intensity, pain-related work interference, physical exertion during work, number of pain regions, and maximal pain intensity. Secondary outcomes: MSP- related sickness absence, self-efficacy, employee involvement | Multicomponent | 20-week participatory ergonomics, comprising three training workshops. The control group were exposed to the usual care | Musculoskeletal-related sickness absence was significantly reduced in the intervention than the control group (-0.48 days per month (95% CI; -0.8– -0.10). Other outcomes were not significantly affected. |
| 3 | Jalalvandi et al. (2022) (16) | Exercise as a form of ergonomics | RCT | Healthcare workers in Iran, including nurses with chronic non-specific LBP (n = 22) and TENs (n = 22) | Low back pain, pain and disability scores | Multicomponent | Two therapeutic methods of back exercises and transcutaneous electrical nerve stimulation (TENS) | Pain and disability scores decreased significantly in both groups (*P*-value < 0.001) compared to the baseline values. The TENS group recorded a significant reduction in pain scores and disability scores compared to the back exercise group after adjusting for the baseline values. |
| 4 | Mercè Soler-Font et al. (2019) (19) | Prevention and management of MSP | CRCT | Nursing staff (eight clusters including 473 nurses and nursing aides)  Spain | Musculoskeletal pain, work functioning, and sickness absence | Multicomponent | Three evidence-based components: participatory ergonomics, health promotion activities and case management. The control group received the usual occupational health care | The intervention group recorded a significantly lower risk of neck, shoulders and upper back pain (OR = 0.37; 95%CI = 0.14–0.96) at 12 months post-intervention compared to the control. No significant difference was detected between sickness absence and work functioning. |
| 5 | Yang et al. (2021) (28) | Multifaceted ergonomics | CRCT | Intensive care nurses in China (89 in the intervention and 101 in the control) | Report rate of MSDs in past 7 days | Multi-component | A multidimensional intervention programme to improve risk perception, a safe working environment and health behaviour training. The control group was exposed to routine specialist training | A significant improvement in the report rate of WRMDs in the past 7 days was observed in the intervention group after 6 months (OR = 1.953, p = 0.037), application of health behaviour (OR = 0.025, p < 0.001), risk perception (OR = 0.517, p < 0.001), and perception of a safe working environment (OR = 1.637, p = 0.024). |
| 6 | Nguyen et al. (2022) (29) | Exercise and educational ergonomics | Quasi-experimental | Nurses (n = 162 in the intervention and 128 in the control.  Vietnam | Musculoskeletal disorders | Multicomponent | The intervention includes 3 components: training on MSDs, ergonomics training, and instructions for physical exercise. | The prevalence of MSDs in the last 7 days decreased significantly in the intervention relative to the control group, particularly in 4 pain regions: shoulder/upper arm, neck, lower back and hand/wrists. |
| 7 | Pereira et al. (2019) (30) | Effects of multifaceted ergonomics on MSP and sickness leave | CRCT | Office workers, including nurses (763 participants from 14 organisations allocated to 100 clusters)  Australia | Neck pain, sickness absenteeism | Multicomponent | A combination of workstation ergonomics and either 12 weeks of health promotion information or workplace neck-specific exercises | At 12 months, the exercise group displayed lower (p = 0.007) monetized productivity loss, sickness absenteeism and presenteeism relative to the health promotion information group. Likewise, sickness absenteeism was significantly lower in exercise participants |
| 8 | Chaiprateep et al. (2022) (31) | Exercise and educational ergonomics | Quasi-experimental | Nurses (28 in the training group and 30 in the control)  Thailand | Lower back pain | Single | The intervention group received a back exercise programme, focussing on back extension, pelvic tilting, and the knee to the chest for a minimum of 3 days/week for 12 weeks. The control group engaged in normal daily activities | The intervention group displayed significant improvement in pain scores and the Thai version of the Oswestry questionnaire to the control group. Beneficial effects improved significantly during the exercise time-points |
| 9 | Suni et al. (2018) (32) | Exercise as a form of ergonomics | Blinded four-arm RCT | Operating room nurses with non-specific LBP  Four groups: control, counselling, exercise, and a combination of neuromuscular exercise and back care counselling; (N = 219 nurses)  Country: Finland | The intensity of low back pain, physical activity-related fear, work-related fear of pain, sickness absenteeism | Single vs multicomponent | Neuromuscular exercise and back care counselling | The combined arm reflected a significant decrease in LBP intensity after 12 months (p = 0.006; effect size 0.70, CI 0.23-1.17) and pain interfering with work (p = 0.011) relative to the control group. Both combined and exercise-only groups recorded a significant reduction in work-related fear of pain. Only the latter group showed lower physical activity-related fear (p = 0.008).  The combined arm recorded the lowest mean total costs and mean number of sickness absence days (p = 0.025). However, none of the interventions was cost-effective for sick absenteeism. |
| 10 | Higuchi et al. (2022) (33) | Individual online physical therapy | RCT | A total of 120 NCW aged 20 years or older will be randomly assigned to an online individualised therapy group (ITG) or usual group (UG) after obtaining informed consent. | Low back pain | Single | ITG participants received professional advice on LBP and musculoskeletal problems from a physical therapist via online interview and email as often as they wish over a 6-month period. The UG participants only had access to brochures and video feeds related to LBP and fall prevention. | The mean score for LBP was significantly lower in the individualised therapy group compared to the control group based on the ODI score. |
| 11 | Jakobsen et al. (2019) (34) | Participatory organisational intervention | RCT | Healthcare workers were recruited randomly from 27 departments from five hospitals in Denmark. The participatory intervention involved 14 clusters, n = 316 healthcare workers) or a control group (13 clusters, n = 309). | Primary outcome: Use of necessary AD (using digital counters  Secondary outcome: general use of AD, LBP intensity, and work-related back injuries during patient transfer at baseline, 6 and 12 months. | Single | The intervention entailed 2×2-hour workshops with the hospital’s health and safety staff and managers and 2–5 healthcare workers from each department. | At the 12-month follow-up, no significant change was observed in the primary outcome and LBP and back injuries between the intervention and control groups. Nevertheless, the general use of AD measured with accelerometers improved significantly in the intervention than in the control group. |
| 12 | Al-Qaisi et al. (2020) (35) | Ergonomic interventions for performing lateral transfers of patients in operating rooms. | Pre and post-intervention experiment | 16 nursing students and 16 college students were enrolled as nurses and patients, respectively. | Main outcomes were EMG activities and muscular function. | Single | Flat and tilted types of table angles were evaluated, while the 3 conditions involved either a standard blanket sheet, a slide board or a plastic bag | Tilted table technique was effective in replacing the physical efforts that the pushing-nurse would have exerted. Significant reduction in the pulling nurse muscles’ EMG activities (p < 0.05). Tilted table was favoured in terms of subjective Borg-ratings, but this device required more time (7.22 seconds) than the flat table for complete transfer of patients (P< 0.05). Findings depicted available ergonomic interventions for performing lateral transfers, without exerting substantial muscle activities among nurses. |
| 13 | Sezgin & Esin, (2018) (36) | An ergonomic intervention designed based on behavioural change model to prevent WMSDs among intensive care unit nurses. | Pre-test post-test design | 72 nurses from two hospitals. | Rapid Upper Risk Assessment Form (RULA) scores and pain intensity | Single | Nurse-delivered Ergonomic Risk Management Program (ERMP) was conducted followed by 26-week follow-up. Assessment included risk reports, RULA scores, and ICU environment assessment. | Nurses experienced a significant decrease in RULA scores during patient repositioning movements and bending down as 0.82 and 1.40, respectively. Significant decrease in pain intensity and RULA ergonomic risk scores, whereas exercise frequency increased significantly. |
| 14 | Beyan et al. (2020) (37) | Efficacy of multifaceted ergonomics intervention in reducing WMSDs. | Pre-test post-test design | Intervention group (n = 27), control group (CG) (n = 23) | MSD scores, sick leave | Single | Ergonomic interventions were broadly divided into four categories: individual-based such as training; stretching exercises and motivation meetings; engineering interventions such as lifting and usage of auxiliary devices, and administrative intervention: daily 10 min stretching exercises break. | At the 18^th^ month, mean WMSD and sick leave scores increased significantly in both groups (p< 0.001), but no significant interaction was observed. Individual-level interventions were likely to eliminate manual patient lifting by nurses. Administrative measures are strongly recommended. |
| 15 | Bettina Wulff Risør et al. (2017) (38) | Efficacy of patient-handling equipment in improving WMSDs, sick leaves and work-related accidents. | Pre-test post-test design | Intervention and control wards at two hospitals. | MSD reports and sick leaves | Single |  | WMSD problems and sick leaves/days of absence were not significantly influenced by the intervention at 12 month follow-up. However, nurses exhibited more positive attitudes and behaviours for safe patient-handling. |
| 16 | Imai et al. (2021) (39) | Effects of individual-based ergonomic intervention on work performance in healthcare workers. presenteeism and improve productivity. | RCT  104 medical workers randomized into intervention and control |  | Presenteeism, pain intensity, physical and psychological stress, | Multicomponent | Intervention group received a six-month plan of exercise and pain neuroscience education (PNE), which was tailored to individual healthcare workers, including nurses. Control group received only a general feedback post-questionnaire completion. | Significant improvement in presenteeism and pain intensity, with between-group differences in presenteeism post-intervention (*P* < .05). |
| 17 | Marshall et al. (2019) (40) | Effects of kinesiology intervention program on WMSDs | RCT with historical control  Intervention (n= 59), control (n =104) |  | WMSD rates | Multicomponent | The intervention group included injured employees referred to the kinesiology service and followed their treatment program. The multifactorial interventions entailed equipment intervention, no-lift policy, education. Individualised intervention was performed post-primary WMSD  The control group included injured employees that were not referred to the kinesiologists, or who chose not to participate in the program. | A significant reduction in subsequent acute cases of WMSD was observed in the intervention group relative to the control. |
| 18 | Taulaniemi et al. (2019) (41) | Efficacy of a 6-month neuromuscular exercise in addressing low back pain among nurses | RCT  Four groups comprising 219 healthcare workers affected with non-specific LBP (exercise, counselling, combined exercise and counselling, control). A secondary analysis comparing exercisers (n = 110) vs non-exercisers (n = 109). |  | Intensity of low back pain | Single | Exercise was performed twice a week (60 min) in three progressive stages focusing on controlling the neutral spine posture.  Secondary outcomes: pain interfering with work, lumbar movement control, fitness components, and work-related measurements. | Intervention group demonstrated significant reduction in LBP (p = 0.047), and work-interfering pain (p = 0.046), abdominal strength (p = 0.033), improved lumbar movement control (p = 0.042), and physical functioning in heavy nursing duties (p = 0.007). |
| 19 | Hosseini et al. (2022) (42) | Effects of Nursing Stretch Break (NSB) application in reducing musculoskeletal complications among nurses | Pre- and post-intervention study.  71 nurses in governmental hospitals.  Iran | WMSDs prevalence in all body regions |  | Single | The intervention comprised 8 main menus, including registration, stretches, user panel, questions from the researcher, about us, contact us, reports, and answers to questions | Excluding elbows and knees, the WMSDs symptoms in the last 7 days reduced significantly in all nurses’ body parts post-intervention. The intensity of WMSD pain also reduced in all body parts, except the |

Note: WMSD = work-related musculoskeletal disorders, LBP = low back pain, RCT = randomised control trial, CRCT = cluster randomised control trial, OR = odds ratio, CI = confidence interval

WMSD = work-related musculoskeletal disorders, RCT = randomised control trials, CRCT = cross-over randomised control trials, MSP = musculoskeletal pain
